# Geoeffective jets impacting the magnetopause are very common

**DOI:** 10.1002/2016JA022534

**Published:** 2016-04-23

**Authors:** F. Plaschke, H. Hietala, V. Angelopoulos, R. Nakamura

**Affiliations:** ^1^ Space Research InstituteAustrian Academy of SciencesGrazAustria; ^2^ Institute of Geophysics and Planetary PhysicsUniversity of CaliforniaLos AngelesCaliforniaUSA

**Keywords:** magnetosheath, jets, scales, magnetopause, geoeffective

## Abstract

The subsolar magnetosheath is penetrated by transient enhancements in dynamic pressure. These enhancements, also called high‐speed jets, can propagate to the magnetopause, causing large‐amplitude yet localized boundary indentations on impact. Possible downstream consequences of these impacts are, e.g., local magnetopause reconnection, impulsive penetration of magnetosheath plasma into the magnetosphere, inner magnetospheric and boundary surface waves, drop outs and other variations in radiation belt electron populations, ionospheric flow enhancements, and magnetic field variations observed on the ground. Consequently, jets can be geoeffective. The extend of their geoeffectiveness is influenced by the amount of mass, momentum, and energy they transport, i.e., by how large they are. Their overall importance in the framework of solar wind‐magnetosphere coupling is determined by how often jets of geoeffective size hit the dayside magnetopause. In this paper, we calculate such jet impact rates for the first time. From a large data set of Time History of Events and Macroscale Interactions during Substorms (THEMIS) multispacecraft jet observations, we find distributions of scale sizes perpendicular and parallel to the direction of jet propagation. They are well modeled by an exponential function with characteristic scales of 1.34*R*_E_ (perpendicular) and 0.71*R*_E_ (parallel direction), respectively. Using the distribution of perpendicular scale sizes, we derive an impact rate of jets with cross‐sectional diameters larger than 2*R*_E_ on a reference area of about $100\;R_{E}^{2}$ of the subsolar magnetopause. That rate is about 3 per hour in general, and about 9 per hour under low interplanetary magnetic field cone angle conditions (<30°), which are favorable for jet occurrence in the subsolar magnetosheath.

## Key Points

- First determination of magnetosheath high‐speed jet cross‐sectional scale size distribution
- First determination of geoeffective jet impact rates on the subsolar magnetopause
- Impact rates of large jets are much higher than occurrence rates of other dayside transients

1

 

## 1 Introduction

**Figure 1:**
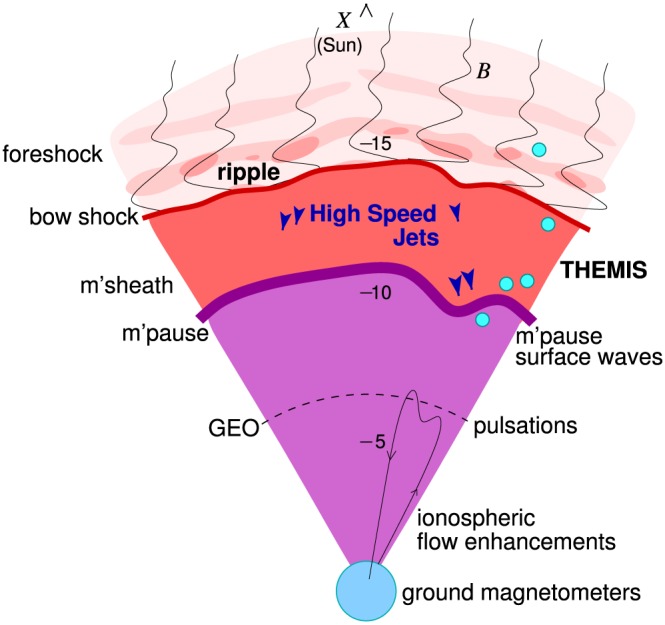
Sketch illustrating the relationship between magnetosheath high‐speed jets and their various effects.

At the dayside bow shock, the solar wind plasma is decelerated from supermagnetosonic to submagnetosonic speeds [e.g., *Spreiter et al.*, 1966]. Behind that boundary, the plasma is denser but much slower than in the pristine solar wind. The plasma's dynamic pressure is typically lower by almost an order of magnitude but may transiently reach or even surpass upstream solar wind values within coherent high‐speed jets [e.g., *Plaschke et al.*, 2013, and references therein]. As illustrated in Figure 1, these jets can propagate toward the magnetopause, impinge on it, cause large amplitude boundary indentations on impact [e.g., *Shue et al.*, 2009; *Amata et al.*, 2011], possibly trigger local reconnection [*Hietala et al.*, 2012], or cross the magnetopause via impulsive penetration [e.g., *Gunell et al.*, 2012; *Dmitriev and Suvorova*, 2015]. As a result, magnetospheric compressional waves or magnetopause boundary surface waves may be generated [see *Plaschke et al.*, 2009]. These waves, in turn, may excite field line resonances [e.g., *Southwood*, 1974] and/or affect radiation belt electrons by modifying their drift path or removing them by magnetopause shadowing [e.g., *Elkington et al.*, 2003; *Turner et al.*, 2012]. Jet impacts can be directly observable from ground, e.g., in the form of localized ionospheric flow enhancements and magnetic field variations [*Hietala et al.*, 2012]. Hence, jets can be geoeffective.

Magnetosheath high‐speed jets are important because of this potential geoeffectiveness, because of the energy and momentum that the jets are able to deposit in the magnetosphere. The downstream consequences of jets depend on the amounts of mass, momentum, and energy that they transport. Ultimately, these quantities are related to the scale sizes of jets, parallel and perpendicular to their propagation directions, which we denote henceforth with *D*_∥_ and *D*_⊥_. Here *D* stands for diameter.

Both *D*_∥_ and *D*_⊥_ are related to the mechanisms that generate jets. Localized jets are expected to result from a rippled quasi‐parallel bow shock [*Hietala et al.*, 2009, 2012; *Hietala and Plaschke*, 2013], from hot flow anomalies [*Savin et al.*, 2012] and foreshock bubbles [*Archer et al.*, 2015], or from magnetic reconnection inside the magnetosheath [e.g., *Retinò et al.*, 2007]. Global scale dynamic pressure enhancements (with very large *D*_⊥_) may be generated by discontinuities in the interplanetary magnetic field (IMF) interacting with the shock and/or with back‐streaming ions in the foreshock region [*Lin et al.*, 1996a, 1996b; *Archer et al.*, 2012; *Dmitriev and Suvorova*, 2012]. This expectation, however, might not agree with observations, as shown by *Archer et al.* [2012]: Although they see jets to be related to IMF discontinuities, they still find them to be rather localized. If the jets are produced by bow shock ripples, then *D*_⊥_ should correspond to the scale sizes of the shock ripples [see *Hietala et al.*, 2009; *Hietala and Plaschke*, 2013]. Recent studies by *Plaschke et al.* [2013] and *Hietala and Plaschke* [2013] and recent simulations by *Karimabadi et al.* [2014] and *Hao et al.* [2016] suggest that rippling of the quasi‐parallel bow shock is responsible for a significant fraction of the jets observed in the dayside magnetosheath. In agreement therewith, *Karlsson et al.* [2015] find “fast paramagnetic magnetosheath plasmoids,” which are closely related to jets, not to be already present in the solar wind, but to be generated at the bow shock or within the magnetosheath. They hypothesize that these “plasmoids” may be foreshock short large‐amplitude magnetic structures [*Schwartz and Burgess*, 1991] that penetrate with high velocity into the magnetosheath through a rippled bow shock.

Jet scale sizes have been determined or estimated in a number of case studies. Typical *D*_∥_ are found to be on the order of 1*R*_E_ [*Němeček et al.*, 1998; *Savin et al.*, 2008; *Archer et al.*, 2012]. *Gunell et al.* [2014] report 5*R*_E_ as the upper limit of *D*_∥_. Using multispacecraft observations, *Hietala et al.* [2012] estimate *D*_⊥_ of jets generated by bow shock ripples to be on the order of a few *R*_E_ and less than 6*R*_E_, whereas *Archer et al.* [2012] find *D*_⊥_ between 0.2 and 0.5*R*_E_ for jets originating from IMF discontinuities. In agreement with both studies, *Gunell et al.* [2014] obtain a value of 7.2*R*_E_ as the upper limit of *D*_⊥_. *Karlsson et al.* [2012] find scale sizes between 0.1 and 10*R*_E_ for a small set of 16 jets, which they call “fast plasmoids.” Distributions of *D*_∥_ have been determined, e.g., by *Plaschke et al.* [2013], who obtain 4000 km as the median value of a distribution of jet scale sizes in the GSE (geocentric solar ecliptic) *x* direction, which roughly corresponds to the flow direction of the jets in their data set.

A distribution of perpendicular scale sizes *D*_⊥_ has not yet been determined. This task is difficult for two reasons: First, a scale size distribution needs to be based on a large data set of jets. Data sets of sufficient sample size have only recently been established [*Archer and Horbury*, 2013; *Plaschke et al.*, 2013]. Second, determining *D*_⊥_ is much more difficult than determining *D*_∥_; the latter can be relatively easily obtained by temporal integration of flow velocities measured by a single spacecraft. For *D*_⊥_, however, simultaneous multispacecraft observations of jets and/or of their environment are required. Here we overcome both difficulties by using the extensive jet data set introduced in *Plaschke et al.* [2013] to obtain, for the first time, a distribution of perpendicular scale sizes *D*_⊥_ of jets observed in the subsolar magnetosheath. Using this distribution, we determine how often jets of geoeffective size hit the dayside, subsolar magnetopause.

## 2 Data and Jet Selection

This study is based on data sets of magnetosheath intervals and jets introduced in *Plaschke et al.* [2013]. That means that we use here exactly the same data and the same selection criteria for magnetosheath intervals and jets as *Plaschke et al.* [2013]. This section is a summary of section 2 of that earlier paper.

Magnetosheath measurements are selected from four years (2008–2011) of data from the five identically instrumented Time History of Events and Macroscale Interactions during Substorms (THEMIS) spacecraft [*Angelopoulos*, 2008]. They are complemented by solar wind measurements from NASA's OMNI high‐resolution data set [*King and Papitashvili*, 2005]. Time intervals are preselected during which a THEMIS spacecraft was within 7 to 18*R*_E_ of the Earth's center, in a 30° wide cone around the GSE *x* axis, centered at Earth and open toward the Sun. The latter criterion restricts THEMIS measurements to ±2 h around local noon.

From the preselected intervals, magnetosheath intervals are selected as follows: (1) Ion densities measured by the THEMIS Electrostatic Analyzers (ESA) [*McFadden et al.*, 2008] are required to be at least twice as large as in the solar wind, where solar wind measurements for a specific point in time are given by averages of OMNI data from the preceding 5 min. (2) The ESA‐measured omnidirectional energy flux of 1 keV ions must be larger than that of 10 keV ions. (3) Magnetosheath intervals must be longer than 2 min and (4) all quantities of interest (THEMIS ESA ion moments, Flux‐Gate Magnetometer (FGM) measurements [*Auster et al.*, 2008], and solar wind magnetic field and ion moments) must be available. Application of all these criteria yields 6960 intervals of 2736.9 total hours of magnetosheath and solar wind data.

Within these intervals, jet intervals are selected as follows: (1) The dynamic pressure in the GSE *x* direction ( $p_{d,x}=\rho v_{x}^{2}$) must surpass one quarter of the solar wind's dynamic pressure (*p*_d,*x*_>*p*_d,sw_/4) over the entire jet interval of length *t*_jet_. Here *ρ* and *v*_*x*_ are the ion mass density (assuming protons only) and the velocity in the GSE *x* direction. (2) At least once within that interval, *p*_d,*x*_>*p*_d,sw_/2 must hold; the time of maximum dynamic pressure ratio *p*_d,*x*_/*p*_d,sw_ is denoted by *t*_0_. (3) One minute long intervals before/after jet intervals are called prejet and postjet intervals; all should lie within a magnetosheath interval as defined above. (4) The velocity *v*_*x*_ must be negative throughout the jet intervals and (5) return to values above *v*_*x*_(*t*_0_)/2 within prejet and postjet intervals. By applying all of these criteria, 2859 jets are selected. More details on the selection process can be found in section 2 of *Plaschke et al.* [2013].

## 3 Multispacecraft Jet Observations

Each of the 2859 jets is identified in measurements of one of the five THEMIS spacecraft, which we denote as the reference spacecraft for that particular jet. To determine *D*_⊥_ of jets, observations by at least one more spacecraft near the reference spacecraft are required. We determine whether a second THEMIS spacecraft was simultaneously present in the magnetosheath in a plane perpendicular to the jet flow direction. In particular, we require the angle between the vectors

(1) $$\vec{d}={\vec{r}}_{\text{second}}-{\vec{r}}_{\text{reference}}$$

and $\vec{v}$ to be in the 80° to 100° range at times *t*_0_, where $\vec{r}$ denotes spacecraft positions and $\vec{v}$ the ion velocity measured at the reference spacecraft. Furthermore, the second spacecraft must be in the magnetosheath at least over the interval ±(*t*_jet_+1min) around *t*_0_ to ensure that the jet seen by the reference spacecraft may not remain undetected by the second spacecraft because *t*_0_ is too close to an end of its magnetosheath dwell time. Here *t*_jet_ and *t*_0_ pertain to the reference spacecraft; they are defined in the previous section 2.

Applying these criteria, we obtain a set of 662 cases of jet observations by a reference spacecraft and context providing observations in the sheath by a second spacecraft, positioned in a plane perpendicular to the respective jet flow direction at the reference spacecraft. These cases are associated with 561 of the 2859 single‐spacecraft jet observations, as for 101 of those, two additional spacecraft provide context to a jet observation by a reference spacecraft. We refer to the 662 case collection as the 2SC (two spacecraft) data set.

Table 1 shows how the 662 cases in the 2SC data set trace back to the different THEMIS spacecraft. Apparently, almost exclusively the inner THEMIS spacecraft (THA, THD, and THE) are involved. The reason is their orbital configuration between 2008 and 2011: The apogee distances of their orbits ranged between 11 and 13*R*_E_, i.e., their apogees were in the magnetosheath when in the subsolar sector, and much closer to Earth than the apogees of THB and THC. Furthermore, the apogee passing times were roughly synchronized. Consequently, when jets were observed by any of the three inner THEMIS spacecraft, close to apogee, the other two spacecraft were likely to be nearby. It is, hence, not surprising that combinations of reference and second spacecraft involve almost only the inner THEMIS spacecraft.

**Table 1:** 2SC Data Set Separated Into Spacecraft Combinations^a^

Second	Reference Spacecraft:				
Spacecraft:	THA	THB	THC	THD	THE
THA	‐	1	0	100	95
THB	0	‐	0	0	0
THC	0	1	‐	1	3
THD	84	0	0	‐	155
THE	82	0	1	139	‐

a Numbers in the table add up to 662, the number of cases in the 2SC data set.

## 4 Perpendicular Scale Sizes

A *D*_⊥_ distribution can be statistically determined from the probability *P*_s_ of jet observation by the second spacecraft at different distances $d=|\vec{d}|$ from the reference spacecraft (equation (1)). Jets with diameters smaller than *d* cannot be observed at the second spacecraft, and jets of larger size will only be observed with a certain nonvanishing probability.

**Figure 2:**
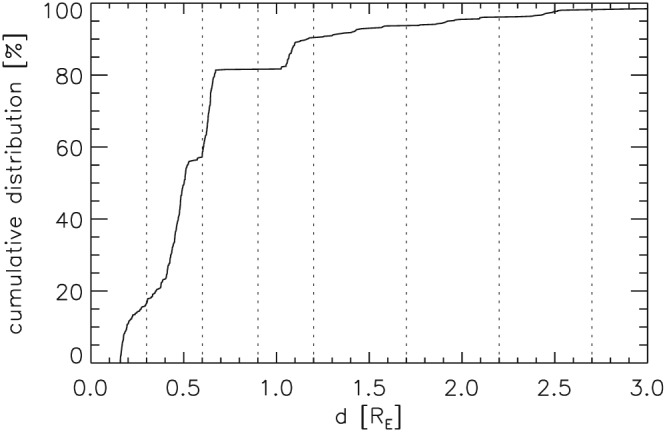
Cumulative distribution of distances *d* in the 2SC data set. Dotted lines illustrate selected *d* ranges.

The cumulative distribution of spacecraft distances *d* in the 662 cases of the 2SC data set is depicted in Figure 2. Certain distances (larger slope) are more common than others. The minimum and maximum distances are 0.16 and 5.45*R*_E_, respectively. In 651 out of 662 cases (over 98%) distances *d* are below 3*R*_E_ and in 539 cases (over 81%) below 0.7*R*_E_.

To compute *P*_s_ as a function of *d*, we categorize the 662 cases of the 2SC data set with respect to *d*. Eight ranges in *d* selected in accordance with Figure 2 are depicted by dotted lines in that figure and defined in the second column of Table 2. As distances *d* are not equally distributed within the ranges, the average distances $\bar{d}$ (third column) are not range centered. Between *N* = 270 and 5 cases fall into the corresponding ranges. We define that a second spacecraft observes the same jet if *t*_0_ as obtained from reference spacecraft measurements lies within a jet time interval (*t*_jet_) computed from the second spacecraft measurements. This will only be the case for a subset *N*_s_ of cases per range, which yields the probability *P*_s_=*N*_s_/*N*. As can be seen in the last column of Table 2, the probability *P*_s_ of reference and second spacecraft detecting jets simultaneously diminishes from 63% at $\bar{d}=0.2\;R_{E}$ to essentially zero, about 3*R*_E_ away from the reference observations.

**Table 2:** *P*_s_ as a Function of $\bar{d}$ a

Range	*d* Range	Average *d*:	# Cases:	# Sec. Obs.:	*P*_s_
*i*	(*R*_E_)	$\bar{d}$ (*R*_E_)	*N*	*N*_s_	(%)
1	0.0–0.3	0.20	111	70	63%
2	0.3–0.6	0.45	270	102	38%
3	0.6–0.9	0.64	159	44	28%
4	0.9–1.2	1.08	58	12	21%
5	1.2–1.7	1.40	22	1	5%
6	1.7–2.2	1.94	16	0	0%
7	2.2–2.7	2.45	13	1	8%
8	2.7–3.2	2.96	5	0	0%

a Columns show ranges in *d* and corresponding average distances $\bar{d}$, numbers of cases *N* with *d* in corresponding ranges, numbers of cases *N*_s_ thereof in which simultaneous jet observations took place by the second spacecraft, and corresponding probabilities *P*_s_ in percent.

**Figure 3:**
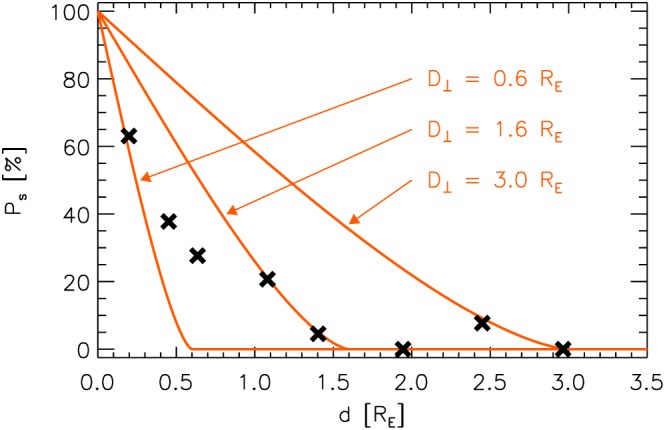
Black crosses: $P_{s}(\bar{d})$ determined from 2SC data set observations. Red lines: Expected probabilities *P*_s,1_ assuming jets of unique $D_{⊥}=0.6\;R_{E}$, $D_{⊥}=1.6\;R_{E}$, and $D_{⊥}=3.0\;R_{E}$, after equation (2).

The probabilities $P_{s}(\bar{d})$ are depicted by black crosses in Figure 3. Using these probabilities, we can check whether a single *D*_⊥_ suffices to explain the $P_{s}(\bar{d})$. If we assume that jets have a circular transverse cross section of fixed diameter *D*_⊥_, then *P*_s_ should be well modeled by the following function:

(2) $$P_{s,1}=\left\{\begin{array}{cc} \frac{2}{\pi }\arccos\left(\frac{d}{D_{⊥}}\right)-\frac{2d}{\pi \overset{2}{\underset{⊥}{D}}}\sqrt{\overset{2}{\underset{⊥}{D}}-d^{2}}, & \text{for}\;d<D_{⊥}\\ 0, & \text{for}\;d\geq D_{⊥}. \end{array}\right.$$

This analytical probability model has been recently used by *Gunell et al.* [2014] to estimate upper limits for transverse scale sizes of jets. Its derivation can be found in appendix A. In Figure 3, functions *P*_s,1_(*d*), after equation (2), for different *D*_⊥_ are represented by red lines.

Apparently, jets of a single *D*_⊥_ cannot explain $P_{s}(\bar{d})$ as determined from observations. If $D_{⊥}=0.6\;R_{E}$ is chosen, *P*_s_ from range 1 ( $\bar{d}=0.2\;R_{E}$) may be explained by equation (2), but then *P*_s,1_=0 would follow for *d* > 0.6*R*_E_, which disagrees with observations. In contrast, jets of *D*_⊥_=3.0*R*_E_ could explain the probabilities obtained for the highest ranges 7 and 8, but then the model would significantly overestimate *P*_s_ for smaller distances *d*. Hence, jets should be characterized by a distribution of different perpendicular scale sizes *D*_⊥_.

Such a probability distribution function may be given by an exponential function of the following form:

(3) $$P_{⊥}=\frac{1}{D_{⊥0}}e^{-D_{⊥}/D_{⊥0}},$$

where *D*_⊥0_ is a characteristic transverse diameter and *P*_⊥_ is a function of *D*_⊥_. As can be seen,

(4) $$\overset{\infty }{\underset{0}{\int }}P_{⊥}(D_{⊥})\;dD_{⊥}=1.$$

The probability of a second spacecraft observing jets, as a function of the distance *d* from the reference spacecraft, is then given by

(5) $$P_{s,m}(d)=\overset{\infty }{\underset{D_{⊥}=d}{\int }}P_{s,1}(d,D_{⊥})\;P_{⊥}(D_{⊥})\;dD_{⊥},$$

where *P*_s,1_(*d*,*D*_⊥_) is given by equation (2), case *d* < *D*_⊥_. Unfortunately, there is no analytic solution to the integral (5); it must be solved numerically.

**Figure 4:**
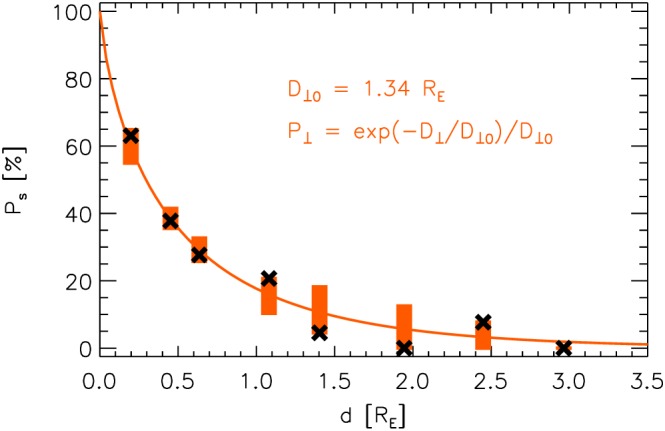
Black crosses: $P_{s}(\bar{d})$ as in Figure 3. Red line: *P*_s,m_(*d*) after equation (5) with *D*_⊥0_=1.34*R*_E_. The red bars enclose the 15.9 and 84.1 percentiles of the binomial distributions for the model‐predicted probabilities $P_{s,m}(\bar{d})$ and the numbers *N* of cases associated with the different ranges/distances $\bar{d}$ (see also Tables 2 and 3).

In equation (3), *D*_⊥0_ is a free parameter that must be determined from observations, more precisely from $P_{s}(\bar{d})$ as given in Table 2. Therefore, we minimize the sum of squared differences between modeled *P*_s,m_ and observed *P*_s_ per range (number *i*), i.e., per distance ${\bar{d}}_{i}$, weighted by the numbers of cases *N*_*i*_ from the 2SC data set contributing to that range:

(6) $$\frac{\underset{i}{\sum }\left({\left(P_{s,m}({\bar{d}}_{i})-P_{s}({\bar{d}}_{i})\right)}^{2}N_{i}\right)}{\underset{i}{\sum }N_{i}}=\min,$$

where *P*_s,m_ is given by equation (5) and $P_{s}({\bar{d}}_{i})$ can be found in the *i*‐th row of the last column of Table 2. As a result of the minimization, we obtain: *D*_⊥0_ = 1.34*R*_E_. Therewith, we are able to compute *P*_s,m_(*d*). This function is depicted by a red line in Figure 4, which also shows observational $P_{s}(\bar{d})$ values (black crosses).

As can be seen in the figure, the modeled values $P_{s,m}(\bar{d})$ come quite close to those actually observed: $P_{s}(\bar{d})$. These latter values should lie within the intervals covered by the red bars in that figure with a probability of at least 68.2% (corresponding to ±1 standard deviation around the mean). The bars enclose the 15.9 and 84.1 percentiles of the binomial distributions for the model‐predicted probabilities $P_{s,m}(\bar{d})$ and the numbers *N* of cases associated with the ranges. Upper and lower percentiles of the binomial distributions and expected numbers of cases of second spacecraft jet observations (*N*_s,m_) that are covered by the red bars in Figure 4 are given in Table 3. Notably, all the observed numbers *N*_s_ lie between the upper and lower *N*_s,m_, which confirms the good agreement between observed probabilities *P*_s_ and modeled values *P*_s,m_ that are based on the distribution given by equation (3) and *D*_⊥0_=1.34*R*_E_.

**Table 3:** Expected Numbers of Cases^a^

Range	Lower		# Sec. Obs.	Upper	
*i*	Percentile	*N*_s,m_	*N*_s_	Percentile	*N*_s,m_
1	13%	61	70	88%	72
2	15%	96	102	86%	112
3	16%	41	44	86%	52
4	8%	6	12	88%	12
5	8%	1	1	92%	4
6	0%	0	0	94%	2
7	0%	0	1	93%	1
8	0%	0	0	91%	0

a Columns show range number, Figure 4 lower percentiles and corresponding numbers of cases *N*_s,m_ of jet observations by the second spacecraft, actual numbers observed *N*_s_, upper percentiles and corresponding *N*_s,m_.

It should be noted that the *D*_⊥_ distribution found in this section is based on a subset of all jets that occurred, while a THEMIS spacecraft was in the magnetosheath; the subset comprises only jets that were observed by a spacecraft. However, jets of smaller *D*_⊥_ are more likely to be missed by a spacecraft; hence, they do not contribute as much to the distribution as larger jets. Consequently, we expect small *D*_⊥_ jets to be underrepresented in this distribution. The difference between the *D*_⊥_ distribution of observed jets derived here and the true *D*_⊥_ distribution of all occurring jets is important for the jet impact rates computed in section 6.

## 5 Parallel Scale Sizes

For comparison, we shall also calculate a distribution of *D*_∥_ equivalent to *P*_⊥_. An estimate of *D*_∥_ can be obtained for every jet observation by integrating the ion velocity in the direction of $\vec{v}(t_{0})$ (flow parallel direction at *t*_0_) over the jet time interval of duration *t*_jet_:

(7) $$D_{∥}=\underset{t_{\text{jet}}}{\int }\frac{\vec{v}(t_{0})\cdot \vec{v}(t)}{\left|\vec{v}(t_{0})\right|}\;dt.$$

**Figure 5:**
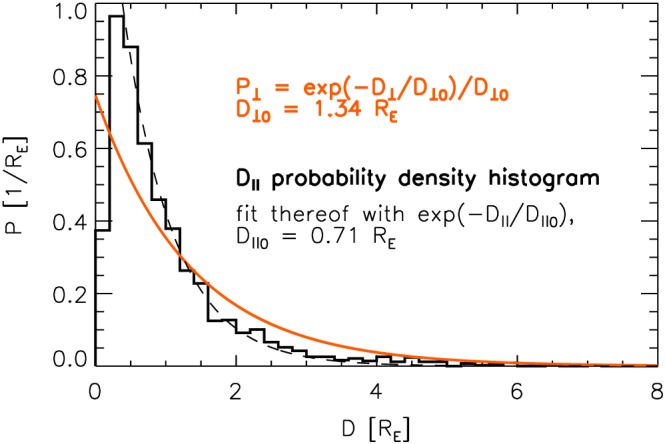
Red line: *P*_⊥_(*D*_⊥_) after equation (3) with *D*_⊥0_=1.34*R*_E_. Black histogram: probability density of *D*_∥_ estimates based on jet observations by THA, THD, and THE. Dashed black line: Fit $∼\exp(-D_{∥}/D_{∥0})$ to this histogram between 0.4 and 8.0*R*_E_, resulting in *D*_∥0_=0.71*R*_E_.

To compute a *P*_∥_ distribution, we could use *D*_∥_ estimates from all 2859 jets selected by *Plaschke et al.* [2013]. However, in this case a comparison of *P*_∥_ with *P*_⊥_ would not be straightforward; the latter function is essentially based on measurements from the inner THEMIS spacecraft, THA, THD, and THE, the orbits of which are similar to each other but differ significantly from the orbits of the outer THEMIS spacecraft, THB and THC. Thus, for determining *P*_∥_, we will use only *D*_∥_ estimates from 2126 single‐spacecraft jet measurements by THA, THD, and THE. The probability density distribution of these estimates is shown by a black line in Figure 5. *P*_⊥_(*D*_⊥_) is depicted by a red line, for comparison.

Apparently, the occurrence probability of lowest *D*_∥_ jets (below 0.2*R*_E_) is very small. This is due to the spin period time resolution of the THEMIS data (3s), which constitutes the smallest observable jet time interval. Assuming typical velocities of >100 km/s, we obtain a minimum *D*_∥_>300km≈0.05*R*_E_. The detection of jets with *D*_∥_ on that order will be impeded, resulting in a notable underestimation of the occurrence of the shortest jets.

With the exception of these jets, the probability density distribution of *D*_∥_ seems to be well approximated by an exponential function of the form $∼\exp(-D_{∥}/D_{∥0})$. Fitting this function to the histogram between *D*_∥_=0.4*R*_E_ and 8*R*_E_ yields *D*_∥0_=0.71*R*_E_. The fit is depicted by a dashed line in Figure 5, which matches the histogram remarkably well. We infer that the probability density function *P*_∥_ should be of the same type as equation (3):

(8) $$P_{∥}=\frac{1}{D_{∥0}}e^{-D_{∥}/D_{∥0}}$$

with *D*_∥0_=0.71*R*_E_. What we note at the end of section 4 also applies to *P*_∥_: The distribution is based solely on jets that were observed by a THEMIS spacecraft. Hence, it is biased toward jets of larger *D*_⊥_ (cross section).

## 6 Jet Impact Rate

To determine the impact rate of geoeffective jets on the dayside subsolar magnetopause we need: (1) the distribution of observed jet perpendicular scale sizes *P*_⊥_ (see section 4); (2) the rate of jet observations *Q*_obs_; and (3) a unitless correction factor *C* to account for jets that are not observed by a spacecraft but should be occurring and impacting the magnetopause. Taking all that into account, the general equation to compute the number of jets impacting on the dayside subsolar magnetopause per unit time (*Q*_imp_), of perpendicular size *D*_⊥min_ or larger is given by

(9) $$Q_{\text{imp}}=\overset{\infty }{\underset{D_{⊥\min}}{\int }}C\;P_{⊥}\;Q_{\text{obs}}\;dD_{⊥}.$$

Here *C* and *P*_⊥_ (given by equation (3)) are functions of *D*_⊥_, and *Q*_obs_ may be computed as a function of the upstream conditions.

We determine *Q*_obs_ from the jet and magnetosheath data set compiled by *Plaschke et al.* [2013]. In principle, the rate is given by the quotient of the number of jets observed by a spacecraft while in the magnetosheath and the total time spent in the sheath by that spacecraft. For consistency, we only use THA, THD, and THE observations. The total time of subsolar magnetosheath data from these three spacecraft is 2387.8 h. During this time, they observed in total 2126 jets. Hence, the rate of jet observations by a single spacecraft near the dayside subsolar magnetopause is

(10) $$Q_{\text{obs}}=\frac{2126}{2387.8\;h}=0.89\;h^{-1}=2.5\cdot 10^{-4}\;s^{-1}.$$

Note that *Q*_obs_ is a general jet observation rate independent of upstream conditions. Furthermore, this rate is not equivalent to the median jet recurrence time of only 140 s reported by *Plaschke et al.* [2013]. The 140 s is the median length of intervals between subsequent jet observations. As jets sometimes occur in series, the median recurrence time is much shorter than the time that can be derived via $Q_{\text{obs}}^{-1}=4043\;s$.

Under low IMF cone angle conditions (≤30°), during which jet occurrence is greatly enhanced, the magnetosheath observation time by THA, THD, and THE is reduced to 235.2 h. During that time, 682 jets are observed, yielding a 3 times higher rate of jet observations $Q_{\text{obs},\leq 30^{\circ }}=2.90\;h^{-1}=8.1\cdot 10^{-4}\;s^{-1}$.

**Figure 6:**
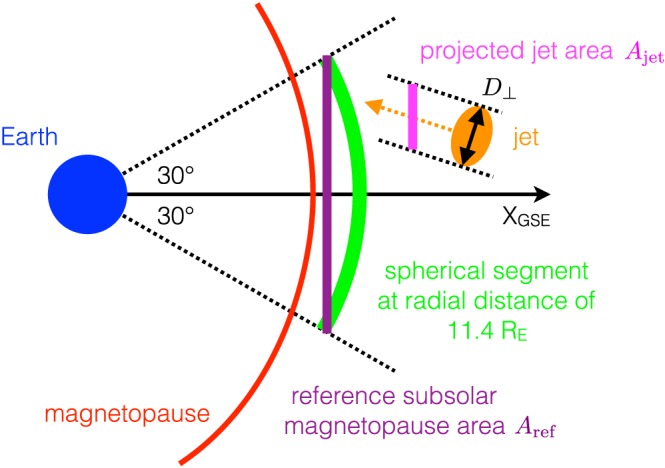
Sketch illustrating the reference and jet areas *A*_ref_ and *A*_jet_ perpendicular to the GSE *x* direction.

The correction factor *C* is, in principle, the quotient of a reference area, over which we would like to count jet impacts, and the cross‐sectional area of the jets (Figure 6):

(11) $$C=\frac{A_{\text{ref}}}{A_{\text{jet}}}.$$

The reference area should be derived from the size of the subsolar magnetopause via $A_{\text{ref}}∼D_{\text{mp}}^{2}$, and the jet area should be proportional to the squared perpendicular scale size $A_{\text{jet}}∼D_{⊥}^{2}$. Then *C* ^−1^ gives the probability that a single spacecraft situated at a point within *A*_ref_ would observe a jet of cross section *A*_jet_ passing through *A*_ref_. Hence, the impact rate of jets of a certain size *A*_jet_ onto *A*_ref_ is *C* times larger than the rate of observations of jets of the same size. This is expressed in equation (9).

The jets selected as detailed in section 2 are characterized by an enhanced dynamic pressure along the negative GSE *x* direction. Therefore, the propagation/flow directions of the jets are mainly aligned with −*x*. Thus, the jets' cross‐sectional planes are mostly perpendicular to the *x* direction. It makes sense to define both areas *A*_ref_ and *A*_jet_ so that they are also perpendicular to the Earth‐Sun line.

As shown in Figure 6, following the preselection criteria outlined in section 2, we define *A*_ref_ as the plane perpendicular to *x* that is bound by (1) a 30° wide cone around *x* open toward the Sun and (2) the radial distance of 11.4*R*_E_, which is the average distance from Earth of the subsolar magnetosheath observations by THA, THD, and THE. As can be seen in the sketch of Figure 6, that circular area covers a large part of the subsolar magnetopause. Its size is

(12) $$A_{\text{ref}}=\pi \;{\left(11.4\;R_{E}\;\sin30^{\circ }\right)}^{2}=102\;R_{E}^{2}.$$

Furthermore, *A*_jet_ is given by the jets' cross section $\pi D_{⊥}^{2}/4$ projected onto *A*_ref_. As illustrated in the figure, that projected area depends on the angle between the jet propagation/flow direction and the negative GSE *x* direction, which we denote as *θ*. We obtain

(13) $$A_{\text{jet}}=\frac{\pi \;D_{⊥}^{2}}{4\;\cos\theta }.$$

The projected jet area is larger than its cross‐sectional area. A mean angle *θ* can be obtained from the *Plaschke et al.* [2013] jet data set by averaging over angles $\arccos(-v_{x}(t_{0})/|\vec{v}(t_{0})|)$. From jet observations by THA, THD, and THE, we obtain *θ* = 25°. That value also holds when considering only jets observed under low IMF cone angle conditions (≤30°).

With (4), (11), and (13), equation (9) becomes:

(14) $$\begin{array}{cc} Q_{\text{imp}} & =\frac{4\;A_{\text{ref}}\;\cos\theta \;Q_{\text{obs}}}{\pi \;D_{⊥0}}\overset{\infty }{\underset{D_{⊥\min}}{\int }}e^{-D_{⊥}/D_{⊥0}}\frac{dD_{⊥}}{D_{⊥}^{2}}\\ =\frac{4\;A_{\text{ref}}\;\cos\theta \;Q_{\text{obs}}}{\pi \;D_{⊥0}\;D_{⊥\min}}\;E_{2}(D_{⊥\min}/D_{⊥0}), \end{array}$$

where *E*_2_ is the exponential integral function of order 2. The integral limit *D*_⊥min_ sets the minimum size of jets under consideration. Setting this limit to *D*_⊥min_=2*R*_E_ to represent geoeffective jets and using (10), we obtain

(15) $$Q_{\text{imp}}=2.9\;h^{-1}.$$

When using $Q_{\text{obs},\leq 30^{\circ }}=2.90\;h^{-1}$ (low IMF cone angle conditions), that rate increases by a factor of 3 to

(16) $$Q_{\text{imp},\leq 30^{\circ }}=9.4\;h^{-1}.$$

It should be noted that *Q*_imp_ is proportional to the reference plane *A*_ref_, defining the size of the dayside subsolar magnetopause, and to the rate of jet observations *Q*_obs_, which is 3 times larger under low IMF cone angle conditions than in general.

## 7 Discussion

In section 4, we find perpendicular scale sizes to be distributed in accordance to equation (3) with *D*_⊥0_ = 1.34*R*_E_. The median size ${\bar{D}}_{⊥}$ is given by

(17) $$\overset{{\bar{D}}_{⊥}}{\underset{0}{\int }}\frac{1}{D_{⊥0}}e^{-D_{⊥}/D_{⊥0}}\;dD_{⊥}=-e^{-{\bar{D}}_{⊥}/D_{⊥0}}+1=\frac{1}{2}$$

which yields

(18) $${\bar{D}}_{⊥}=\ln(2)D_{⊥0}=0.93\;R_{E}.$$

*Hietala et al.* [2012] and *Archer et al.* [2012] obtain perpendicular scale sizes *D*_⊥_ of a few *R*_E_ and between 0.2 and 0.5*R*_E_, respectively; ${\bar{D}}_{⊥}$ is midway between those previously reported values. It also lies within the range between 0.1 and 10*R*_E_ stated by *Karlsson et al.* [2012] and fulfills the upper limit of 7.2*R*_E_ reported by *Gunell et al.* [2014]. As equation (3) is a distribution function yielding nonzero probabilities for all possible *D*_⊥_ (from 0 to ∞), a certain number of jets, slightly less than 0.5%, is predicted to be larger than 7.2*R*_E_. Moreover, although ${\bar{D}}_{⊥}$ is not within the 0.2 and 0.5*R*_E_ range reported by *Archer et al.* [2012], our distribution predicts that a significant fraction (17%) of jets will have *D*_⊥_ within that range.

A few assumptions are made to obtain the probability distribution function of observed *D*_⊥_. First, an exponential function (equation (3)) is chosen as a model. A similar function matches the *D*_∥_ distribution remarkably well, and by using equation (3) the multispacecraft based probabilities *P*_s_ are also well reproduced. Both facts strengthen our confidence in the choice of (3). Nevertheless, another (similar) function may reproduce the observations equally well.

Second, we assume that simultaneous observations of jets by second spacecraft are related to the same jets seen by the reference spacecraft. That does not have to be always true. Two jets of smaller cross section propagating next to each other may also result in simultaneous jet observations by the reference and second spacecraft. Consequently, *D*_⊥0_ = 1.34*R*_E_ might be slightly too high. By contrast, even if jets are large enough to pass over both reference and second spacecraft, (1) they may not have been identified in the data from the second spacecraft, e.g., because the second spacecraft was too close to crossing the magnetopause or (2) *t*_0_ of the reference spacecraft may not be within the corresponding jet interval as obtained from the second spacecraft measurements, e.g., because *t*_jet_ is short. These cases are treated as if the jet were not observed by the second spacecraft. Consequently, *D*_⊥0_ = 1.34*R*_E_ might be slightly too low. In summary, there are reasons to believe that *D*_⊥0_ could be slightly lower or higher than determined, but it is unclear which of the discussed effects dominates, and if any of them is able to affect significantly the result of *D*_⊥0_.

Third, we assume the perpendicular cross section of jets to be circular, which also need not be the case. The magnetic field direction could break the symmetry and lead to two distinct distributions for *D*_⊥_ [see *Karlsson et al.*, 2012]. Determining those distributions would, however, require an even larger set of jet observations and hence is out of the scope of this paper.

As noted at the end of section 4, *P*_⊥_ is only a distribution of observed jet scale sizes. Hence, small *D*_⊥_ are underrepresented. This effect may be corrected by the factor *C*, derived in section 6. Hence, the actual distribution of jet scale sizes should be proportional to

(19) $$C\;e^{-D_{⊥}/D_{⊥0}}∼\frac{1}{D_{⊥}^{2}}e^{-D_{⊥}/D_{⊥0}}.$$

The occurrence of jets of very small perpendicular scales appears to be very high, to the point that the expressions given above cannot be normalized any more: Their integration starting from 0 yields ∞, as jets of very low cross‐sectional area are extremely unlikely to be observed by a spacecraft. Unfortunately, observational limitations such as the available spacecraft separations do not allow us to resolve small scales well enough to constrain fits with different functional forms. The new Magnetospheric Multiscale mission [*Burch et al.*, 2015], however, should be able to shed light into the small scale end of the jet distribution, which is of great interest to studies of impulsive penetration, as that mechanism should only work for plasma structures that feature widths below a few ion gyro‐radii [*Brenning et al.*, 2005].

As we do not know the relationship between cross sections and parallel scale sizes of jets, *P*_∥_ cannot even be corrected. Nevertheless, we can compare *P*_⊥_ and *P*_∥_. As evidenced by $D_{⊥0}=1.34\;R_{E}>D_{∥0}=0.71\;R_{E}$, the observed jet parallel scale sizes tend to be significantly smaller than the jet perpendicular or cross‐sectional scale sizes. Following equation (18), we find a median diameter along jets of ${\bar{D}}_{∥}=0.49\;R_{E}$ that is about half as large as ${\bar{D}}_{⊥}$. If we assume jets to feature approximately the same aspect ratio (small/large *D*_⊥_ corresponding with small/large *D*_∥_), then they should be flattened rather than elongated in propagation direction, looking like pancakes flying flat side first. This picture of jets contrasts global magnetospheric simulation results by *Karimabadi et al.* [2014]. As depicted in Figure 15 of their paper, jets appear to be elongated, meandering structures. How the observational and simulation results can be reconciled is yet to be determined. It should also be noted that the term “jet” is usually associated with an elongated structure. Hence, in the light of our findings, that term does not appear to be fully suited to describe the observed plasma structures.

Furthermore, ${\bar{D}}_{∥}=0.49\;R_{E}$ is lower than typical *D*_∥_ of about 1*R*_E_ reported, e.g., by *Němeček et al.* [1998], *Savin et al.* [2008], and *Archer et al.* [2012]. It is also lower than the median *D*_∥_ of 4000km = 0.63*R*_E_ stated in *Plaschke et al.* [2013]. This apparent discrepancy may be (at least partly) explained by the fact that the model function (8) assigns a higher probability of occurrence to jets of lowest *D*_∥_ size (see black solid and dotted lines in Figure 5). In addition, *Gunell et al.* [2014] report a median upper limit of 5*R*_E_ for *D*_∥_. Again, we obtain a probability of slightly less than 0.5% for jets to have a larger *D*_∥_ than 5*R*_E_, confirming the limit set by *Gunell et al.* [2014].

In section 6 we obtain equation (14) for the rate at which jets of a certain minimum size hit the dayside magnetopause. The values we input to that equation are derived from THEMIS observations near the ecliptic plane. Hence, we assume that there are no latitudinal variations in the jet occurrence and properties. Furthermore, the occurrence rate of jets is largest close to the nominal bow shock location [*Plaschke et al.*, 2013], implying both that their source is near the shock and that many of the jets do not make it all the way to the magnetopause. In the current study, however, we use almost exclusively the jet observations of the three inner THEMIS spacecraft located near the nominal magnetopause. Thus, the impact calculations appropriately consider only jets that come close to the magnetopause.

For the following reasons, we consider a diameter of *D*_⊥min_=2*R*_E_ to be reasonable for jets to be considered geoffective: (1) Using THEMIS multispacecraft measurements, *Shue et al.* [2009] find that a jet of that size caused a significant magnetopause indentation of about 1*R*_E_. (2) *Hietala et al.* [2012] report that jets with *D*_⊥_ between 1.5 and 2*R*_E_ (Cluster interspacecraft separation) caused magnetic pulsations at geostationary orbit, localized ionospheric flow enhancements, and corresponding signatures in ground magnetometer measurements. We find that such geoeffective jets hit the subsolar magnetopause (reference area of $102\;R_{E}^{2}$) about 3 times per hour, in general, and about 9 times per hour during favorable, low IMF cone angle conditions [see *Plaschke et al.*, 2013]. This is consistent with the observations by *Hietala et al.* [2012] and *Hartinger et al.* [2013] of waves at geostationary orbit indicating almost continuous bombardment of the magnetosphere during long, continuous intervals of quasi‐radial IMF. It is also worth noting that the impact rate *Q*_imp_ of these large jets alone is about three times as large as the overall (all jet sizes) single‐spacecraft observational occurrence rate *Q*_obs_.

Finally, we can compare *Q*_imp_ to the (observational) occurrence rates of geoeffective foreshock transients. According to THEMIS statistics, hot flow anomalies occur about once every 2 h and foreshock bubbles only about once per day under favorable, high solar wind speed conditions [*Turner et al.*, 2013]. Furthermore, the impact rate of geoeffective jets is much larger than, for example, the well‐known occurrence rate of substorms, once every 2 to 3 h under favorable, southward IMF conditions [e.g., *Jackman et al.*, 2014].

## 8 Summary and Conclusions

We have used a data set of magnetosheath high‐speed jets based on THEMIS measurements and compiled by *Plaschke et al.* [2013] to investigate how many jets of geoeffective size hit the dayside subsolar magnetopause per unit time. Statistical results from multispacecraft observations of jets are well modeled when assuming an exponential distribution of observed perpendicular (cross sectional) scale sizes of jets (equation (3)), using a characteristic size of *D*_⊥0_=1.34*R*_E_. Parallel scale sizes are obtained from single‐spacecraft measurements by velocity integration (equation (7)). Again, observed parallel sizes are found to be exponentially distributed, with a characteristic size of *D*_∥0_=0.71*R*_E_. If we assume the aspect ratio to be approximately the same for all jets, then they should all resemble pancakes flying flat side first. This finding contrasts simulation results by *Karimabadi et al.* [2014], who obtain jets that are rather elongated in the direction of propagation, meandering through the magnetosheath. Furthermore, in the light of this finding, the term “jet” does not appear to be fully suited to describe the observed plasma structures.

The parallel and perpendicular distributions pertain to observed jets. However, jets of smaller cross section are less likely to be observed. After correcting for that effect and taking into account the rate of observation of jets by a single spacecraft *Q*_obs_, we compute, for the first time, how many jets of a certain size hit a reference area of the subsolar magnetopause per unit time. We find that geoeffective jets of cross‐sectional diameter larger than 2*R*_E_ should hit the subsolar magnetopause about 3 times per hour under general conditions and about 9 times per hour under favorable, low IMF cone angle conditions. These impact rates are much higher than the reported occurrence rates of other dayside transients. Consequently, magnetosheath high‐speed jets must be considered as an important transmitter of energy and momentum from the solar wind into the inner magnetosphere.

## Appendix A Probability Model for Jet Observations With Two Spacecraft

### A.1

We assume that jets have a circular cross section of diameter *D*_⊥_ transverse to their flow/propagation direction. The variable *a* shall denote the distance from the center of the jet's cross‐sectional area to the location at which the reference spacecraft crosses that area. Necessarily, *a*≤*D*_⊥_/2. The differential probability of the reference spacecraft crossing the cross‐sectional area at a distance *a* from its center is given by

(A1) $$dP_{1}=\frac{2\mathrm{\pi a}}{\pi D_{⊥}^{2}/4}da.$$

The second spacecraft shall be located at a distance *d* from the reference spacecraft, also in a plane transverse to the jet flow direction. If we assume the second spacecraft to be located exactly on the edge of the jet, then the law of cosines yields the following relation:

(A2) $$\phi =\arccos\left(\frac{a^{2}+d^{2}-D_{⊥}^{2}/4}{2\mathrm{ad}}\right),$$

where *ϕ* is the angle between a line from the jet's center to the reference spacecraft and a line between the two spacecraft. Keeping *D*_⊥_, *a*, and *d* constant, the second spacecraft would observe the jet for smaller angles *ϕ*, i.e., cross its cross‐sectional area, but not observe the jet for larger angles *ϕ*. Accordingly, we find the following probabilities *P*_2_ for the second spacecraft to observe the jet for fixed values *D*_⊥_, *a*, and *d*:

(A3) $$P_{2}=\left\{\begin{array}{ccc} 1, & \text{for}\;d<D_{⊥}/2, & a<|D_{⊥}/2-d|\\ 0, & \text{for}\;d>D_{⊥}/2, & a<|D_{⊥}/2-d|\\ \phi /\pi , & \text{for}\; & a>|D_{⊥}/2-d| \end{array}\right.$$

with *ϕ* = *ϕ*(*a*,*d*,*D*_⊥_) after equation (A2) being a function of *a*, *d*, and *D*_⊥_. In this study, the distance *a* is an observationally unknown quantity. We can eliminate that quantity by integrating over *P*_2_d*P*_1_, which yields the desired probability *P*_s,1_ of identifying the jet with a second spacecraft, given *d* and *D*_⊥_:

(A4) $$P_{s,1}(d,D_{⊥})=\int P_{2}(a,d,D_{⊥})\;dP_{1}(a,D_{⊥})$$

(A5) $$=\overset{D_{⊥}/2}{\underset{a=0}{\int }}P_{2}(a,d,D_{⊥})\frac{2a}{D_{⊥}^{2}/4}da.$$

This integral can be solved analytically, yielding equation (2) as the solution.
